# How Fatigue Is Experienced and Handled by Female Outpatients with Inflammatory Bowel Disease

**DOI:** 10.1155/2013/153818

**Published:** 2013-09-01

**Authors:** Anne Beck, Palle Bager, Peter Errboe Jensen, Jens F. Dahlerup

**Affiliations:** ^1^Department of Hepatology and Gastroenterology, Aarhus University Hospital, Aarhus, Denmark; ^2^Education of Nurses in Aarhus, VIA University College, Aarhus, Denmark

## Abstract

*Background*. Fatigue is a significant aspect of everyday life for patients with inflammatory bowel disease (IBD), and it influences their health-related quality of life. Little is known about fatigue from the patient's perspective. *Aim*. To investigate how female IBD patients experience and handle fatigue. *Methods*. The study included 11 female outpatients. These patients were 40–59 years old and had IBD ≥ one year and a significantly increased fatigue score. Patients with severe active IBD, anaemia, comorbidity, or pregnancy were excluded. The included patients agreed to participate in a semistructured interview. The interviews were analysed using Malterud's principles of systematic text condensation. *Results*. The patients described physical and mental symptoms of fatigue that led to social-, physical-, and work-related limitations with emotional consequences. To handle fatigue, the patients used planning, priority, acceptance, exercise, and support. Two of the eleven patients used exercise on a regular basis. Surprisingly, some patients indicated that they did not need to talk with professionals about their fatigue unless a cure was available. Conclusion. Fatigue in IBD includes physical and mental symptoms that limit the patients' social-, physical-, and work-related lives. Despite this, some patients expressed that they had chosen to accept their fatigue.

## 1. Background

Inflammatory bowel disease (IBD), comprising Crohn's disease (CD) and ulcerative colitis (UC), is a chronic medical condition with unknown aetiology, but genetic and environmental factors likely have an influence on progression of the disease. The symptoms can include diarrhoea, bloody stools, abdominal pain, and extraintestinal manifestations such as fatigue. Fatigue is a well-known symptom in chronic diseases, including IBD, and it is often connected to complaints and worries among IBD patients [1–3]. Despite the lack of an accepted definition of fatigue, it can be described as *a persistent, overwhelming sense of tiredness, weakness, or exhaustion resulting in a decreased capacity for physical and/or mental work *[4, 5]. Due to the lack of an accepted definition and the fact that fatigue is a subjective and unspecific symptom, it can be a challenge to adopt the right measurement tools for fatigue diagnosis and quantification [4]. This was expressed in a review from 2010 that identified 252 different ways to measure fatigue in chronic diseases, of which 150 have only been used once [6].

Over the last decade the interest in fatigue has increased [7]. However, a review from 2010 by van Langenberg and Gibson reveals that only ten studies were found to include data on fatigue in IBD patients. Of the ten studies, only one study measured fatigue in IBD patients, as its primary endpoint [5].

A recent study of the prevalence of fatigue among IBD patients found that approximately 44% of the patients were fatigued, using the 95th percentile of the score of the general population as a cut off point [4]. Furthermore, this and other studies indicated increased values for fatigue, if disease activity was present [4, 8, 9]. However, it is important to underline that fatigue is also present when the disease is in remission [4]. The study did not find a significant difference in fatigue between the UC and CD patients [4]. Other studies of fatigue among the general population indicate that stratifying for gender and age is necessary. Females in general are characterised by higher fatigue scores than men, which is also the case for IBD patients [4, 10, 11]. Both for the general population and the IBD patients, age and fatigue are correlated. Increased age leads to increased fatigue [4, 10].

Fatigue affects the quality of life for IBD patients, and fatigue leads to increased worries among IBD patients [1–3, 12]. In a study from 2010 on worries and concerns, *energy level *was ranked as the next biggest concern, even above *bowel control *[3].

Despite the increased focus on fatigue and its impact on quality of life, the studies on fatigue in association with IBD remain incomplete. Studies measuring the prevalence of fatigue and studies measuring fatigue in relation to quality of life have been conducted [1–3, 7, 10, 12]. However, studies that describe how patients with IBD experience and handle fatigue are not identifiable by a systematic literature search. A recent review from 2013 confirmed the lack of data from patients' perspective on fatigue and IBD. Twenty-eight studies were reviewed, and none of the studies asked patients to describe the experience of fatigue. Furthermore, the review underlined that methods of managing fatigue need to be further explored [13].

The aim of this study was to investigate how female outpatients with IBD experience and handle fatigue.

## 2. Patients and Methods

### 2.1. Patients

Female patients from the outpatient clinic of the Department of Gastroenterology at Aarhus University Hospital were consecutively screened for participation. The inclusion period was five weeks in the autumn of 2012. All female IBD patients between the ages of 40 and 50 years were prescreened. This subgroup of IBD patients was chosen, as this group has been shown to express the most fatigue [4]. Other inclusion criteria were the ability to talk and understand Danish and an IBD diagnosis for more than one year, as findings suggest that patients improve their management of fatigue over time [14]. Patients with severe active IBD, anaemia, comorbidity, and/or pregnancy were excluded from participation. Disease activity was measured using the Harvey-Bradshaw Index (HBI) and Simple Clinical Colitis Activity Index (SCCAI). Final inclusion required a certain level of fatigue, as evaluated using the Multidimensional Fatigue Inventory (MFI-20). 

MFI-20 is a 20-item, self-reported instrument designed to measure five dimensions of fatigue: general fatigue, physical fatigue, reduced activity, reduced motivation, and mental fatigue. Each dimension contains four questions, and the scores range from 4 to 20. A higher score indicates greater fatigue [15]. The dimensions of greatest importance have been described as *general fatigue* and *physical fatigue* [4, 10, 11]. Therefore, this study focuses on these two dimensions. The cut off points for final inclusion were ≥11 for general fatigue and ≥10 for physical fatigue. These cut off points are the 75th percentile of the score of the general population [10]. 

**Figure 1:**
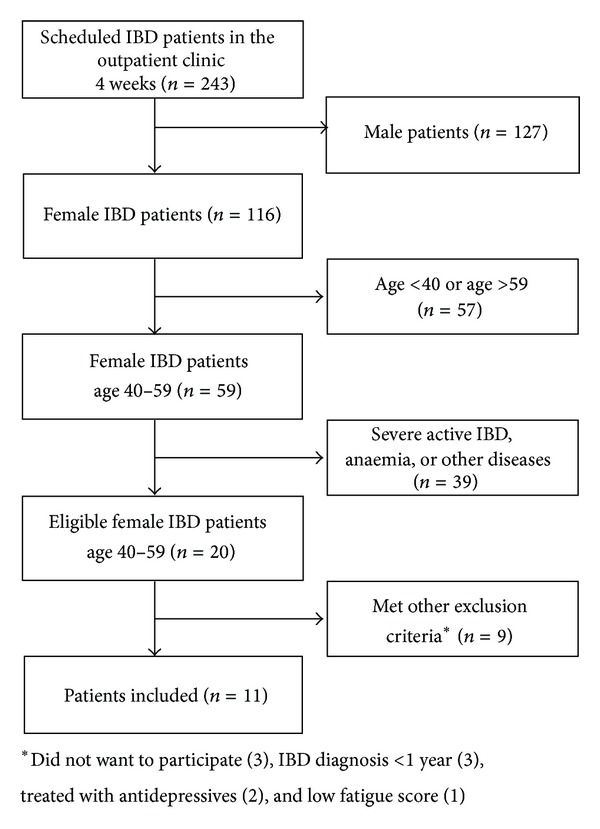
Patient flow.

After the screening process, 11 patients were invited to participate in the study. The patient flow is illustrated in Figure 1. The patients were included in interviews until saturation, where no additional or new data involving fatigue were identified in the interviewing process in accordance with the background theory for interviews [16, 17].  Table 1 shows the demographic characteristics of the 11 patients.

### 2.2. Interviews

All interviews were semistructured interviews and were conducted when the patients had appointments at the outpatient clinic. Before the interviews the patients were informed about the design and the aim of the study. Each interview lasted approximately 30 minutes and was conducted in a private room at the hospital. The investigator performed all interviews. An interview guide containing 5 topics was used to structure the interview (Table 2). The guide was inspired by a study on the experience of fatigue among patients with rheumatoid arthritis (RA). Before the guide was used in the previous study, it was piloted in three interviews and minor amendments were made [18]. The interviews were recorded and transcribed by the investigator. 

### 2.3. Analysis

Each of the recorded interviews was subjected to analysis using Malterud's principles of systematic text condensation. This method is similar to the editing style of Miller and Crabtree [19, 20]. 

The principles of systematic text condensation involves four steps. First, the investigator read the interviews as a whole, attempting to achieve a total impression. Temporary themes were created. Second, the interviews were decontextualised. The investigator identified codes with matching meaning units (quotations or words). These meaning units were the parts of the text that were found to express aspects of importance for understanding how patients with IBD experience and handle living with fatigue. The third step was estimation of the codes, and from these codes, subcodes were identified. Finally, the chosen codes/subcodes were converted into an analytical text, which makes the findings in this study. The interviews were recontextualised to ensure that the investigator was accurate to the source data [20]. The analytical text is based on statements, descriptions, and opinions about fatigue from the interviews. These statements, descriptions, and opinions were selected based on their ability to describe the fatigue phenomenon and to what extent it was important to the patients. Furthermore, the investigator paid special attention to the aspects of fatigue that were repeated throughout the different interviews. 

The codes were presented to and discussed with the coauthors, and some corrections were made. 

### 2.4. Ethical Considerations

The study was presented to the Ethics Committees of Denmark and the Danish Data Protection Agency. No formal approval was needed, but a national law related to personal data protection had to be followed. All patients provided informed verbal consent. All patients were informed about voluntariness, anonymity, confidentiality, publication, and their right to withdraw from the study at any time. The patients were allowed to choose another day for the interview. This allowed the investigator to be sure of the patients' ability to committing themselves thoroughly to the interview. 

## 3. Results

### 3.1. Experience of Fatigue

Three superior codes were identified under the heading *experience of fatigue*, which are named *physical and mental fatigue*,* limitations*, and *emotional consequences.* These three codes were encapsulated in analytical texts in the following paragraphs. 

#### 3.1.1. Physical and Mental Fatigue

Fatigue was initially divided into physical and mental fatigue. The patients used the appellations *physical/bodily fatigue* and *mental fatigue/a feeling of a tired head* themselves and indicated that the two concepts affect each other.

*Physical Fatigue.* Physical fatigue was described as an uncomfortable feeling that could be felt in every joint. This uncomfortable feeling especially expressed itself as heavy limbs. This could contribute to slow movements. Patients described a lack of control when *groping*, as fatigue's effect on movements could affect patients' abilities to walk and to hold on to a glass. This is illustrated in the quotation below:

*“If I ignore it, I will be more and more physically tired and irritated and stressed, and then I can get the feeling that I start dropping things. I start groping and I do not really have proper control. Many glasses have been broken due to that.” *

Some patients believed that there was an association between pain and fatigue. Pain in the joints was particularly emphasised. One patient described becoming so tired that it almost hurt. Another patient explained that she often felt pain in the joints in connection with gardening for instance. This pain contributed to tension in the body, which afterwards entailed an intense fatigue. 

Moreover, the patients mentioned symptoms, such as tightness in the chest, headache, stiffness in the joints, and being sensitive to cold, in relation to fatigue.

*Mental Fatigue.* Mental fatigue was mainly expressed as concentration problems. This could contribute to chaotic thoughts and displacement activities. This was especially a problem during work. Furthermore, patients reported that due to fatigue, it could be difficult to remain focused when listening and answering questions in social contexts. Fatigue and the entailing concentration problems were connected with difficulties with remembering, particularly when reading, as illustrated in the quotation below:

*“If you have to read something for the day next day, right… And then, you are sitting there and fighting, right… And you are also fighting to remember what you are reading. I think that's really annoying”*

Additionally, irritability was a major factor that arose when fatigue was at its worst. This irritability especially affected the closest family and friends. 

#### 3.1.2. Limitations

Physical and mental symptoms on fatigue can produce what the patients referred to as lack of energy, exhaustion, and indisposition, which limited the patients in their everyday lives, whether both socially, physically, or at work.

*Social Limitations.* Fatigue and the lack of energy make it difficult to maintain the same level of social life as previously. Patients noted that they sometimes had to cancel social arrangements, such as staff parties and family reunions. Among other factors, this was because the patients needed to be outgoing, in good form, and attentive when socialising. This is expressed in the following quotation:

*“Then, there was something such as family reunion you get invited to, and oh well… You may think that it is quite of a handful if there are many people and you better be a bit social and in good form.” *

As stated previously, the patients expressed how it could be difficult to concentrate when listening and answering because of fatigue. A patient said that it made her nervous beforehand knowing that she at some point would experience fatigue during social gatherings.

Nonetheless, despite fatigue, some patients uttered that they forced themselves to be attentive in gatherings and did not allow family and friends to suffer due to fatigue-related limitations.

However, these patients did not frequently take initiative to arrange different arrangements, as it consumed too much of their energy, which would contribute to fatigue overpowering them afterwards. Therefore, many patients' social networks were limited to the closest family and friends.

*Physical Limitations.* Physical limitations emerged due to a lack of energy, pain, tensions, and stiffness, and these limitations could contribute to patients deliberately choosing not to exercise, even though they were aware of the generally beneficial effects of exercise. One patient said that she had to quit her gymnastic team, as she did not have enough energy due to fatigue. Moreover, the physical limitations also expressed themselves in everyday activities. In particular, cleaning and gardening were difficult to perform. Thus, the patients lost the will to stay active, as great energy and willpower were necessary. In the following quotation, a patient explains how her physical limitations changed her relationship to gardening:

*“You do not enjoy it as you did before. Before you might enjoy walking around and enjoy doing something, right (gardening). And suddenly it is something that just has to be done, right. And it is just not the same, right.”***

*Work-Related Limitations.* Approximately half of the participating patients were working part-time, were not working, or were on sick leave. Whether these employment statuses were due to the symptoms of IBD in general or fatigue could not be clarified in the interviews, but the lack of energy could have an impact. The patients expressed that working was of great importance, and not being able to work the same amount as before was a negative experience. An aspect of the work-related limitations is illustrated by the following quotation:

*“Of course there is a little sorrow about losing everything you were previously, right. Because working used to mean quite a lot to me. I really… I really had a great career and ehh… It feels like I'm living half of a life now.”*

Patients still working expressed that concentration was an essential problem, which could affect their work efficiency. Furthermore, fatigue could affect the choice of tasks made available to the patient. A patient noted that she was forced to only undertake one-person tasks, as she was no longer able to participate in groups. Another patient said that she was worried about whether fatigue could have an effect on her colleagues' perception of her when she had to leave work earlier than expected or call in sick to meetings. Furthermore, not being able to work also affected the social aspect of the patients' lives. 

#### 3.1.3. Emotional Consequences

As previously stated, fatigue produced limitations that affected the patients' everyday live. These limitations could lead to feelings of anger, frustration, sadness, self-pity, worry, and grief. Furthermore, fatigue was often connected with feelings of a guilty conscience. These feelings arose when fatigue limited the patients' social life. The patients did not always have the energy to socialise with their closest family, and sometimes, they could not take on a responsibility in different tasks. The feeling of a guilty conscience especially arose among patients with children:

*“And often I'm tired when I should sit down and play with her and have a good time or do something else… And then, she has to do something herself, right… I think… I feel bad about that.” *

Powerlessness is another feeling that can emerge in connection with fatigue. This was expressed by the patients as them feeling they were limited in their life. Patients said that the powerlessness could be a product of the fact that there currently is no available cure for fatigue, and the patients had to accept and cope with that fact. Patients used the phrase “*fatigue will catch up to you,*” which indicates that the patients felt that fatigue would limit them at some point during their lives. 

**Figure 2:**
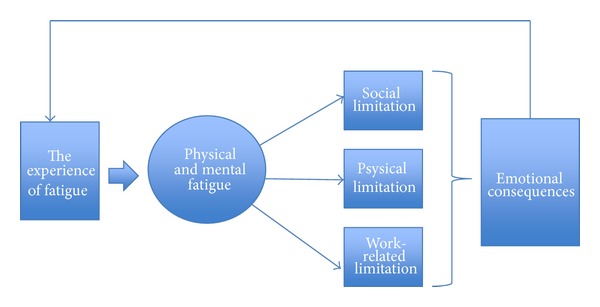
Experience of fatigue in IBD. The experience of fatigue begins with physical and mental symptoms, which leads to social-, physical-, and work-related limitations. These limitations may cause some emotional consequences like bad conscience. The emotional consequences are a part of the experience of fatigue.

Together, the three codes *physical and mental fatigue*, *limitations*, and *emotional consequences *constitute an understanding of how fatigue was experienced among the IBD patients in this study.  Figure 2 shows the connections where symptoms led to limitations, and the limitations led to the feelings discussed above.

### 3.2. Handling of Fatigue

Five superior codes were identified under the title *handling fatigue*.

The five groups were *planning*, *priority*, *acceptance*, *exercise*, *and support. *These codes represent ways to handle fatigue. 

#### 3.2.1. Planning

The patients expressed how planning was used to handle fatigue. This is necessary, as patients only have a certain amount of energy, which should be distributed throughout the whole day.

Planning included a balance between activity and rest.

*Activities.* The first sort of planning involved patients filling up their days with activities. Thus, they attempted to ignore fatigue:

*“It is typically when I do not do anything, then it can knock one out, if you can say so. However, if I'm doing something, then I do not feel it until afterwards.” *

The above quotation emphasises that whilst being occupied, the patient could distance herself from the fatigue and temporarily forget about it. This is possible, as fatigue mostly expresses itself when sitting and relaxing. To distance themselves from fatigue, the patients relied on physical work, for instance, cleaning, gardening, or exercises. The physical work contributed to the patients often using more energy than they had available. Using this method, there was a risk that the patients would use up all their energy and become exhausted. Additionally, the patients noted that fatigue at some point would *catch up to you. *Despite the fact that the patients tried to occupy themselves and forget their fatigue, they still could not completely avoid it.

*Rest and Breaks.* The other component in planning involves structured rests, breaks, relaxation, long night sleeps, and limiting the amount of activities during the day. For instance, some patients had scheduled times for a nap. Other patients described that it was not necessary to take a nap or stated that it was a bad idea due to interruptions in their circadian rhythm, lack of time, or the feeling of being subsequently lethargic. However, a break during the afternoon could be enough to regain energy. The patients relaxed using books, TVs, computers, music, or breathing techniques. The patients described that this break was a necessary requirement for them. However, a patient said that the break limited her day in general, as she had to split up her day. 

The patients who did not take fatigue into account when planning their day said that they tried to listen to their body and take an hour at a time.

#### 3.2.2. Priority

The patients realised that prioritisation was necessary for them, as their total energy was limited. Patients spent their energy on activities of high importance to them. Patients uttered that they reserved energy for their closest family and work, which is illustrated in the following quotation:

*“And I think, I really want to work, right, and therefore I will… Then, there is a priority… I know I have a certain amount of energy, and I use it on the things I like… like my work, right”*

The patients would prioritise activities that would produce joy and meaning. Problems could arise when this prioritised activity turned out to be more energy consuming than the patients had available. Some patients said that they were willing to pay the price and hence have energy shortage, if they could participate in social gatherings that were important to them. 

#### 3.2.3. Acceptance

Despite the feeling of powerlessness that can emerge due to the limitations of fatigue, half of the patients asserted that they had accepted their fatigue. However, this may be some sort of obligatory acceptance, as patients felt they did not have a choice, as there is no cure for fatigue. Patients said that they had to learn to live with fatigue and the reduced level of energy, which is expressed in the following quotation:

*“However, you will just have to accept it. And that contributes to the fact that you will have to stop thinking too much about it and not to dwell on it. At least, that is my strategy”*

However, there were patients who said that they refused to accept the limitations that emerged from fatigue. It could convey that patients forced themselves to participate in social gatherings or physical activities. The lack of acceptance could be due to the previously mentioned priorities, as patients would not accept fatigue when they were dealing with something of importance for them.

#### 3.2.4. Support

Support related to fatigue may come from family and friends, but support can simultaneously come from health professionals.

*Family.* The patients asserted that their closest family members were aware that fatigue was time consuming in everyday life and took this into consideration. For instance, the patients' husbands and boyfriends would aid in childcare, cleaning, and other practical matters. This typically occurred when they could sense that the patients were tired and irritable. In addition, children and husbands were good at determining if the patients needed rest, which the following quotation emphasises:

*“And my foster child, well, he knows that when we come home, we sit down for a while. Well, it is just natural: (I said) ‘Well, but I have to get up… I will rest for a bit', (he replies) ‘Well, then I'll play computer' Well, it is… incorporated into our routine, or what to say. He understands.”*

Nonetheless, patients said that fatigue was not something they talked about in the home, as they did not want to spend too much time discussing the issue during everyday living or simply because they did not feel a need to talk about it. 

Patients without a partner or a supporting network seemed to feel fatigue to a greater extent compared to those living with a partner.

*Health Professionals.* The patients generally did not talk to the health professionals about their fatigue. They did not feel that fatigue was a topic that should be dealt with during their time with the health professionals, even though this study indicates it had a great impact on everyday lives of the patients. The reason for this can among other things be explained by the patients understanding of fatigue as a side effect only, which should not be problematised. Patients' expressed that it was possible to talk about fatigue, but it often required that the patients took initiative themselves to do so. A patient declared that she did not get anything out of talking about fatigue with the health professionals:

*“And if I say that I'm tired, there is a sort of: ‘Yes, it is annoying to be tired'. And I can understand that, because what are they supposed to do? It is not so that we can be drugged with speed or anything…”*

Patients did not feel they needed to talk about fatigue unless the health professionals could somehow treat the problem.

#### 3.2.5. Exercise

Two patients exercised via strength or fitness training several times a week. They both declared that it resulted in them having more energy. As stated previously, patients were aware of the general beneficial effects of exercising, but they did not feel that they had the energy to exercise. This contributed to a vicious circle producing even more fatigue. This is illustrated in the following quotation:

*“Yes, I used to be an active runner. However, I simply do not… Ever since this fall, I have not been able to get up off the shitty couch. Although I know it will give me more energy, and you are never… I have never regretted going for a run, but… However, I just have not been able to get out there.”*

## 4. Discussion

The involvement of health professional intervention related to fatigue among patients with IBD is challenged by this study. For instance, some patients said that they did not have a need to talk about fatigue with health professionals, unless a specific cure for fatigue was available. Despite this statement, this study illuminates that fatigue produces limitations and has emotional consequences, which may dominate the patient's day to a great extent. Therefore, it is important that health professionals focus on fatigue and give the patients the *opportunity* to talk about it. Furthermore, the health professionals must be aware of the risk that the patients' understanding of fatigue (as a side effect only, which should not be problematised) could be a reflection of the health professionals understanding of fatigue. The fact that the health professionals cannot offer a cure or advise can explain why patients feel that the health professionals should not bring up the subject.

The limited frequency of patient exercise was another interesting finding. Only two patients utilized frequently exercised. As exercise has some effect on quality of life for other groups with chronic conditions, it seems obvious that the effect of exercise among IBD patients should be examined to determine how it specifically affects this group and fatigue. 

In this study there was not a focus on specific coping strategies, despite the aim of examining how patients handle fatigue. This is due to the use of Malterud's principles of systematic text condensation, which signify that the investigator must be accurate to the data [20]. Theories about coping were, therefore, not included in this analysis. A pilot study from 2011 dealt with CD patients and fatigue. It suggested two coping strategies (problem solving and solution-focused therapy) that may have had a positive effect on patients with CD [21]. It could be interesting to investigate whether patients with IBD use elements of these coping strategies in their own way to handle fatigue.

Another important question is whether IBD patients experience fatigue differently than other chronic patient groups. A study concerning patients with RA revealed that this patient group among other things experienced pain, stiffness, and heavy limbs. Additionally, the study described that fatigue had a great impact on the patients' everyday life. Anger, frustration, exhaustion, and acceptance were commonly used words in patient interviews [18]. The RA study indicates that patients with RA might experience fatigue in a similar way to IBD patients. Thus, transfer of the results of the present study to other chronic patient groups may be possible. Likewise, the patients' method of handling fatigue can be compared to the same RA study. This patient group also used rest, breaks, relaxation, and planning to handle fatigue. Furthermore, the study indicated that patients had to make a decision about prioritising important activities [18]. This is similar to this study's priority findings. 

This study also indicates that patients without a husband, boyfriend, or social network in general felt fatigue to a greater extent compared to those living with a partner. This was expressed through interviews where the patients without a partner or a supporting social network, perceived it as a cause of fatigue. This is comparable to a study from 2000 by Watt et al. that focused on the general population and described that the patients who lived alone experienced a greater extent of fatigue [11]. 

Other factors than IBD could possibly have had an impact on the present study's findings. For example, one patient was about to be divorced, another had an ill partner, and a third patient had a disabled child. These are all factors that may aggravate fatigue, and they are factors that could have been used to exclude these patients. However, one may argue that these factors establish a realistic picture of reality, as divorce, illness, and other problematic events are part of life. A recent study indicates that fatigue should not be considered only as a direct feature of IBD, as personality variables can explain the intensity of fatigue of IBD patients in remission [22]. This finding emphasises that fatigue is a subjective phenomenon. Therefore it would be difficult to exclude patients with factors that may influence fatigue (such as specific life events and personality), due to the subjectivity of fatigue. 

The findings related to the patients' handling of fatigue have, to some extent, a similarity to the general population's handling of this phenomenon. However, the handling of fatigue among IBD patients may be distinguished from the general population's method. For instance, it was important to focus on how willing patients were to accept consequences when doing something that was energy consuming, as long as it was of great importance. This expresses a great awareness of the consequences of fatigue and its limitations. In addition, IBD patients involved the family in dealing with limitations. The family members demonstrated consideration and took responsibility for certain tasks within their family, as they were aware that fatigue entails a lack of energy. It is dubious that the same family involvement would be observed amongst the general population. However, this is something that requires further investigation to be verified. 

The patients initially filled in all twenty questions in MFI-20 questionnaire (to avoid confusion), but in the investigator's counting of points, focus was on general fatigue and physical fatigue (eight questions). Results from previous studies indicate that these two categories particularly tend towards the highest fatigue scores [4, 10, 11]. During the analysis of the eleven interviews, mental fatigue was determined to be an important factor as well. Therefore, it was necessary to make a subcode named *mental fatigue* in the analysis to provide the best description of how fatigue was experienced by the patients.

This study is hampered by its small sample size, and further research dealing with the perspective of male outpatients is necessary to transfer the results. Another limitation of the study was that the investigator analysed the data single-handed, which increased the risk of being too subjective in the analysis. However, interviews are a subjective form of investigation, and the use of Malterud's principles of systematic text condensation as a method of analysis required the investigator to stay loyal to the source of data. All interviews took place at the hospital, although the environment might not be convenient from the patient's perspective. This might affect the patients' answers. 

## 5. Conclusion

In conclusion, female outpatients with IBD and fatigue experienced physical and mental symptoms related to their fatigue. These symptoms caused some social-, physical-, and work-related limitations that contributed to feelings such as guilty conscience and powerlessness. This describes a connection where symptoms led to limitations, and the limitations led to emotional consequences. The patients used planning, priority, acceptance, and support from their relatives to handle fatigue. Only two patients used exercise on regular basis to achieve more energy. Despite the limitations and the emotional consequences, it is important to accentuate that the patients used acceptance to handle fatigue. Surprisingly, some patients did not express any desire to talk to health professionals about fatigue, unless a cure for fatigue was available.

## Figures and Tables

**Table 1:** Demographic characteristics of included patients.

	Crohn's disease (CD)	Ulcerative colitis (UC)
Number of patients, *n*	7	4
Age mean	45,3	47,5
Disease activity mean (range):		
HBI	5 (2–10)	—
SCCAI	—	1 (0–2)
Location, *n*	L1: 2	E1: 3
	L2: 5	E2: 1
	L3: 2	E3: 0
	L4: 0	
Behaviour of CD, *n*	B1: 5	—
	B2: 1	
	B3: 1	
Treatment, *n*:		
Steroid treatment	0	0
Immunosuppressive treatment	3	1
Biologics treatment	3	1
5 ASA	1	3
None	1	0
Occupation, *n*:		
Work part time	2	1
Work full time	4	2
Retired	1	0
Sick-listed	0	1
Relationship, *n*:		
Living with partner only	1	0
Living with partner and children	4	3
Living with children only	2	1
Living alone	0	0

HBAI: Harvey Bradshaw Activity Index.

SCCAI: Simple Clinical Colitis Activity Index.

L1: terminal ileum, L2: colonic, L3: ileocolonic, and L4: upper digestive tract.

E1: proctitis, E2: left-sided colitis, and E3: Extensive including pancolitis.

B1: nonstricturing-nonpenetrating, B2: stricturing, and B3: penetrating.

**Table 2:** Interview guide for fatigue in IBD: the topics for the semistructured interviews.

(1) How would you describe your fatigue? How does it feel?	
(2) What causes your fatigue?	
(3) How does fatigue affect your daily life? Consequences?	
(4) How do you manage your fatigue?	
(5) How do you experience the professional care for fatigue?	
